# Prevalence of total hepatitis A antibody among 5 to 7 years old children and their mothers in Cambodia

**DOI:** 10.1038/s41598-021-83710-2

**Published:** 2021-02-26

**Authors:** Shintaro Nagashima, Ko Ko, Chikako Yamamoto, E. Bunthen, Serge Ouoba, Channarena Chuon, Masayuki Ohisa, Aya Sugiyama, Tomoyuki Akita, Md. Shafiqul Hossain, Vichit Ork, Bunsoth Mao, Junko Tanaka

**Affiliations:** 1 Department of Epidemiology, Infectious Disease Control and Prevention, Hiroshima University Graduate School of Biomedical and Health Sciences, 1-2-3, Kasumi, Minami-ku, Hiroshima, 734-8551 Japan; 2 Payment Certification Agency, Ministry of Health of Cambodia, Phnom Penh, Cambodia; 3 Unité de Recherche Clinique de Nanoro (URCN), Nanoro, Burkina Faso; 4 Faculty of Medicine, University of Puthisastra, Phnom Penh, Cambodia; 5 Expanded Programme On Immunization, World Health Organization Country Office, Phnom Penh, Cambodia; 6 National Immunization Programme, Ministry of Health, Phnom Penh, Cambodia; 7 University of Health Sciences, Phnom Penh, Cambodia

**Keywords:** Hepatology, Infectious diseases, Viral epidemiology, Disease prevention, Public health

## Abstract

This study determined the prevalence of total hepatitis A antibody (anti-HAV) among 5–7 years old children and their mothers in the whole Cambodia, using a nationwide study, and examined the differences between the two cohorts. A total of 4535 dried blood spot-driven (DBS) samples (2021 mothers and their 2514 children of 5–7 years old) and the concomitant 922 whole blood samples (subset of the whole participants) were collected using a multistage random sampling strategy throughout Cambodia in 2017. Total anti-HAV was detected using the chemiluminescence enzyme immunoassay method. Compared to gold standard whole blood samples, the sensitivity and specificity of DBS mediated anti-HAV detection were 94.8% and 98%, respectively. Total anti-HAV prevalence among mothers was 91.2% (95%CI: 90.0–92.5%), and that of their children was 31.5% (95%CI: 29.7–33.3%). In our study, the low prevalence of total anti-HAV among children indicates the country’s improvement of safe water and food supply, hygiene and sanitation. If the hygiene and sanitation are consistently improved in Cambodia, the prevalence might be no longer increased when the children become adults.

## Introduction

Viral hepatitis A is caused by hepatitis virus A (HAV), a small, naked single-stranded positive RNA virus which can survive on hands and non-porous environments as well as various kinds of foods^1^. Therefore, the transmission route is fecal–oral, and sporadic forms of epidemic outbreaks were reported worldwide. There were also reports on HAV outbreaks among vulnerable groups of people, such as Men who have sex with Men (MSM) and people with intravenous drug use (PWIDs)^2^. Once infected, almost all individuals fully recover with life-long immunity. But the outcome can be fatal, especially if the disease progresses rapidly to fulminant hepatitis.

According to World Health Organization (WHO), hepatitis A caused approximately 7134 deaths in 2016, which accounts for 0.5% of the mortality due to viral hepatitis^3^. The prevalence may vary in each continent or country, but it is closely associated with food and water safety, as well as personal hygiene and sanitation. A multistate outbreak of HAV in the United States in 2016 was reported and all the outbreaks were linked to food-born origin^4^. The outbreak in developing countries had experienced much health burden than developed countries^5,6^.

In Cambodia, there were only a few reports on the prevalence of HAV last decade^7–9^. The increasing trend of HAV prevalence by age was observed, and almost 100% of Cambodian adults were positive for antibodies to hepatitis A virus (anti-HAV) Immunoglobulin G (IgG)^9^. No more studies were conducted for HAV infection in Cambodia since 1998, so that the current disease burden is unexplored. Additionally, the Kingdom of Cambodia is now vehemently moving forwards and the economics, environment, and health care system are improving day by day^10^. Positive economic and societal changes create better sanitation, safe food and water supply, and proper hygiene, leading to a healthy lifestyle. Alongside the development of Cambodia, understanding the trend of infectious diseases is crucial for its specific prevention and control. We expected that anti-HAV prevalence in Cambodia would decrease than before. Thus, this study determined the prevalence of total hepatitis A antibody (anti-HAV) among 5–7 years old children and their mothers in the whole Cambodia, using a nationwide study and examined the differences between the two cohorts.

## Methods

This study is the continuum of our nationwide epidemiological study on the prevalence of hepatitis virus, including HAV, among 5–7 years old children and their mothers in Cambodia. Data collection was conducted in 2017, using a multistage stratified random sampling strategy^11^. The detailed study design procedure and sampling strategy were described in our previously published work^11^. In this study, we report the total anti-HAV prevalence among children and their mothers.

### Subjects

This study included two main groups of subjects.

i. 5–7 years old children who were born between March 2010 and February 2012 and represented the new generation after the country’s gross development
ii. Mothers of the above-mentioned children, who represented the generation before the country’s gross development

Exclusion criteria included children who were not able to give blood because of underlying severe illness or hemophilia, and children whose caregivers did not provide consent for blood sampling.

### Demographic data

Our study included a questionnaire survey designed to explore the immunization status of children, particularly hepatitis B, but the demographic data were also useful for HAV infection. Age, sex, socio-economic status, infectious disease status, and immunization status were extracted and employed to correlate total anti-HAV positivity.

### Collection of blood samples

Two types of sampling techniques were used in this study.

i. Dried blood spot sampling technique using HemaSpot-HF (Spot on Science Inc., Austin, USA) was used to collect the blood samples from all participants by finger prick. Approximately 60 µl (µl) of blood (3 drops) were collected into the HemaSpot-HF device and then kept with a desiccant (silica) at -80˚C until measurement.
ii. Three milliliters (ml) of whole blood samples using venipuncture were collected from randomly selected subgroups of participants (405 mothers and 517 children). The samples were centrifuged, and only the serum was stored at − 80 °C until measurement.

### Detection of total anti-HAV

Total anti-HAV was directly detected from all serum-derived and DBS samples. Measurement was done directly from the serum samples. But DBS driven samples were firstly eluted with an elution buffer at room temperature as previously described^12^. Then, 20 µl elute was mixed with 60 µl of 7.5w/v% albumin D-PBS solution (Wako Pure Chemical Industries, Ltd, Osaka, Japan) before the detection of total anti-HAV.

Total anti-HAV was detected by chemiluminescence enzyme immunoassay method (CLEIA) using VITROS ECiQ Immunodiagnostic System (Ortho Clinical Diagnostics Inc., New Jersey, USA). The resultant C.O.I was interpreted to set the cut-off point for serum sample as follow;

(i) "positive" for total anti-HAV titer < 0.8 C.O.I,
(ii) "borderline" for 1.0 > total anti-HAV titer C.O.I ≥ 0.8 and
(iii) "negative" for total anti-HAV titer ≥ 1.0

### Statistical analysis

To clarify the risk factors of total anti-HAV positivity among children, a multivariate logistic regression analysis using the stepwise selection method (both inclusion and exclusion criteria: *p* < 0.25) was performed among 11 variables of the questionnaires, which included residing area, gender, place of birth, age, mother’s HAV status, mother’s age, mother’s education, house roof, number of siblings, job and traveling time to the health center. For all analyses, *p* < 0.05 was considered statistically significant. Using 922 pairs of serum and DBS samples from same participants, the sensitivity and the specificity of DBS-spotted total anti-HAV detection were calculated against gold standard serum samples. The Receiver Operating Characteristic (ROC) curve was created by scatter plot. All the statistical tests were performed using JMP version 11 (SAS Institute, Cary, NC, USA).

### Ethical consideration

Informed consent was obtained from the mother for her child and herself. If the mother was absent, the father or another caretaker was asked to provide consent for the child. Informed assent was obtained from all children in the study. All specimens and questionnaire data were de-identified, with reference only to a unique identifier. The study protocol was approved by the Cambodia National Ethics Committee for Human Research (392NECHR), the Ethics Review Committee for the WHO Western Pacific Region, and the Ethics Committee for Epidemiology of Hiroshima University (E-573). This activity was reviewed in accordance with the CDC human research protection procedures, and was determined to be human subject research, but CDC involvement did not constitute a direct engagement in human subject research.

## Results

### Subjects

A total of 4535 participants (2514 children and 2021 mothers) were included in the study. DBS samples were collected from each participant, and the serum samples were randomly additionally collected from 922 participants to evaluate the efficacy of DBS for total anti-HAV detection. The detailed characteristics of the enrolled children and their mothers were publicly stated in the previous report^11^. Of the enrolled children, 1270 (50.5%) were male, and the number of 5, 6 and 7 years old children were 1234 (49.1%), 1198 (47.6%) and 82 (3.3%), respectively. The mean age of mothers was 32.5 ± 6.1 years and reported a mean of 2.8 children (medium: 2) with a range of 1–12 children.

### Sensitivity and specificity of total anti-HAV from DBS samples against serum samples

**Figure 1:**
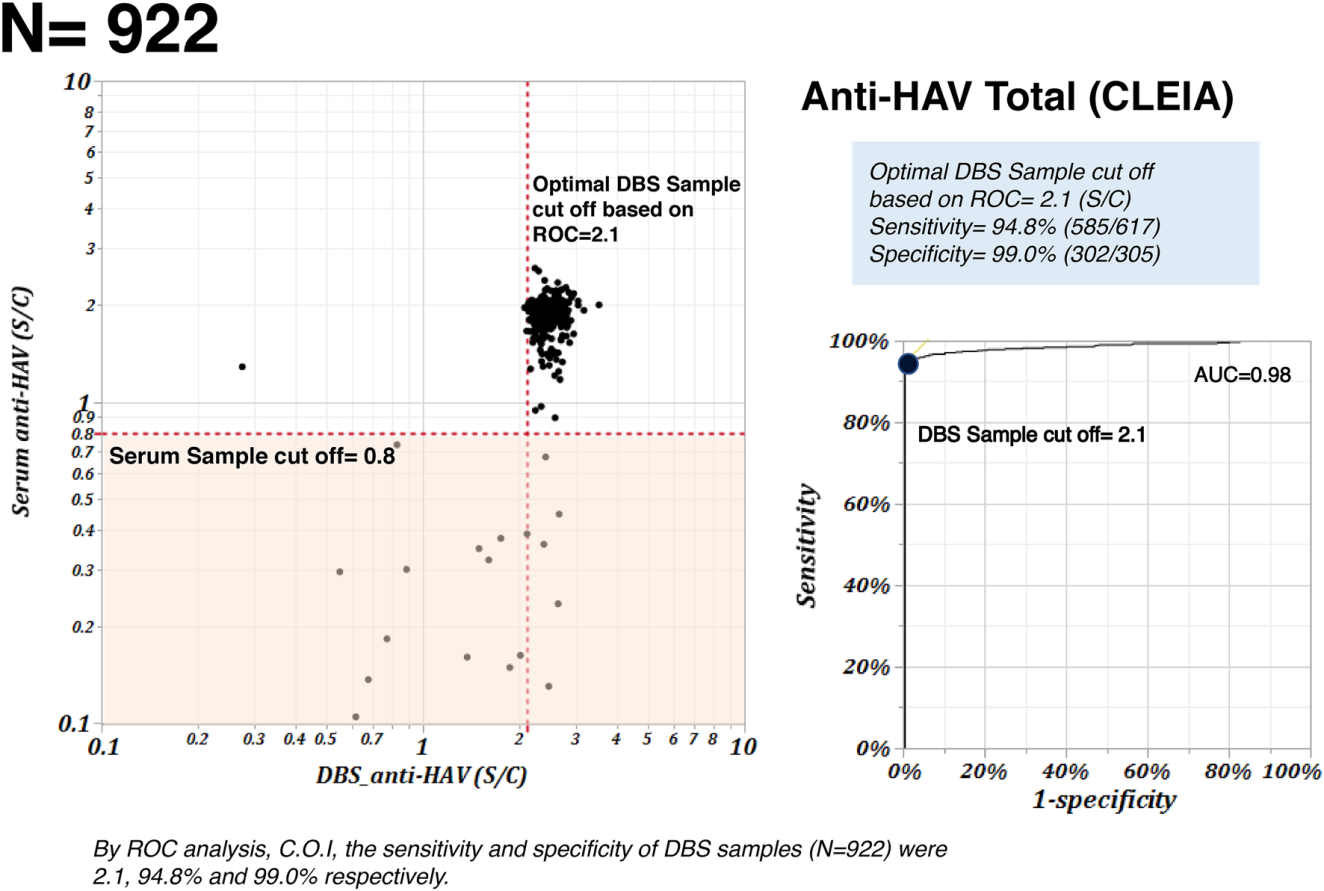
Scatter plot of Serum and DBS sample in Total-HAV Ab. This figure shows the agreement on the measure value of total anti-HAV between dried blood spot sample-driven samples and whole blood samples from 922 randomly selected participants in Cambodia. The scattered plot shows the cut-off value for the detection of total anti-HAV from DBS against the whole blood samples. The area under curve (AUC) was 0.98.

The cut-off index of total anti-HAV titers from serum samples was defined at 0.8. Using 922 serum samples, the sensitivity of DBS-driven samples was 94.8% (508/617), and the specificity was 99.0% (302/305). By mean of ROC analysis, the optimal cut-off index of total anti-HAV using DBS driven samples was set at 2.1 (AUC = 0.98). (Fig. 1).

### Nationwide prevalence of total anti-HAV among children and their mothers

**Figure 2:**
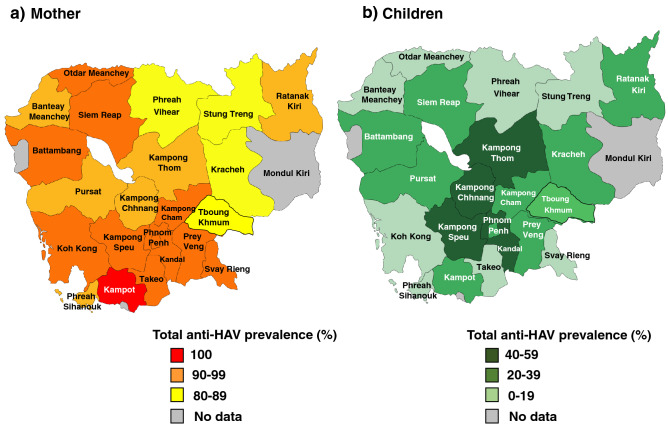
Population density and total anti-HAV prevalence of mother and their child in each province of Cambodia (**a**) represents the map showing total anti-HAV prevalence of mothers in each province of Cambodia in 2017 and similarly (**b**) shows for the children. The intensity of color in each map are changed by its value.

Using the DBS driven samples collected from all enrolled children and their mothers throughout the whole Cambodia, the total anti-HAV prevalence was 31.5% (95%CI: 29.7–33.3%) in children and 91.2% (95%CI: 90.0–92.5%) in mothers. The total anti-HAV prevalence varied by region and ranged from 5.6% to 56.9% among children and 77.6% to 100% among mothers (Table 1). The total anti-HAV prevalence of under 20% among children was found in Stung Treng, Preach Vihear and Otdar Meanchey provinces, followed by Koh Kong, Takeo, Svay Rieng and Banteay Meanchey provinces. The highest prevalence of total anti-HAV among children was found in Kampong Chhang followed by Kampong Speu, Preah Sihanouk, Kampong Thom and Kandal provinces. Meanwhile, the low prevalence among their mothers was found in Kracheh followed by Stung Treng and Preach Vihear provinces, all of which have prevalence under 80%. The highest prevalence of total anti-HAV at 100% among their mothers was found in Kampot followed by prevalence of over 95% in Siem Reap, Tboung Khmum, Kampong Speu, Battambang provinces. (Table 1, Fig. 2a,b).

**Table 1:** Area-specific prevalence of total anti-HAV among children and their mothers in Cambodia.

No	Province	Children				Mothers			
		No	( +)	*P*	95% CI	No	( +)	*P*	95% CI
1	Banteay Meanchey	144	28	19.4	13.0–25.9	87	78	89.7	83.3–96.1
2	Battambang	180	49	27.2	20.7–33.7	140	133	95.0	91.4–98.6
3	Kampong Cham	180	72	40.0	32.8–47.2	154	138	89.6	84.8–94.4
4	Kampong Chhnang	72	41	56.9	45.5–68.4	63	55	87.3	79.1–95.5
5	Kampong Speu	180	82	45.6	38.3–52.8	151	144	95.4	92.0–98.7
6	Kampong Thom	144	63	43.8	35.6–51.9	115	102	88.7	82.9–94.5
7	Kampot	72	15	20.8	11.5–30.2	50	50	100.0	92.9–100.0
8	Kandal	179	76	42.5	35.2–49.7	142	131	92.3	87.9–96.7
9	Koh Kong	72	10	13.9	5.9–21.9	61	59	96.7	92.3–100
10	Kracheh	72	22	30.6	19.9–41.2	67	52	77.6	67.6–87.6
11	Otdar Meanchey	36	3	8.3	0–17.4	19	18	94.7	84.7–100
12	Phnom Penh	177	58	32.8	25.9–39.7	122	111	91.0	85.9–96.1
13	Preah Sihanouk	36	16	44.4	28.2–60.7	32	27	84.4	71.8–97.0
14	Preah Vihear	36	3	8.3	0–17.4	33	26	78.8	64.8–92.7
15	Prey Veng	180	48	26.7	20.2–33.1	141	128	90.8	86.0–95.6
16	Pursat	108	32	29.6	21.0–38.2	103	89	86.4	79.8–93
17	Ratanak Kiri	36	10	27.8	13.1–42.4	34	30	88.2	77.4–99.1
18	Siem Reap	180	70	38.9	31.8–46.0	153	147	96.1	93.0–99.2
19	Stung Treng	36	2	5.6	0–13.0	33	26	78.8	64.8–92.7
20	Svay Rieng	107	17	15.9	9.0–22.8	92	83	90.2	84.1–96.3
21	Takeo	144	25	17.4	11.2–23.5	112	105	93.8	89.3–98.2
22	Tboung Khmum	143	50	35.0	27.1–42.8	117	112	95.7	92.1–99.4
Total		2514	792	31.5	29.7–33.3	2021	1844	91.2	90.0–92.5

### Risk factors of total anti-HAV positivity among children

By multivariate logistic regression analysis, children from urban areas (Adjusted OR: 0.41, 95%CI: 0.26–0.64, *p* = 0.0001) and the type of house roof (Cement, Adjusted OR: 0.40, 95%CI: 0.16–1.00, *p* = 0.0500) had a lower risk of HAV infection. The job of the household head (Fisherman, Adjusted OR: 2.45, 95%CI: 1.47–4.08, *p* = 0.0005) and the travelling time to the health center (30–44 min, Adjusted OR: 1.51, 95%CI: 1.16–1.98, *p* = 0.0023 & 45–49 min, Adjusted OR: 2.41, 95%CI: 1.68–3.47, *p* < 0.0001) had a higher risk of HAV infection. (Table 2).

**Table 2:** Univariate and multivariate analysis on positivity of total anti-HAV among children in Cambodia.

Factors	N	Univariate analysis			Multivariate analysis		
		Anti-HAV ( +)	(%)	*p* value	AOR	95% CI	*p* value
**Category**							
PP	88	29	33.0	** < 0.0001**	1.29	0.80–2.08	0.3014
Urban	183	25	13.7		0.41	0.26–0.64	**0.0001**
Rural	1748	577	33.1		1.00	Reference	
**Gender**							
Male	1045	324	31.0	0.8031			
Female	974	307	31.5				
**Place of birth**							
Public Hospital	405	122	30.1	**0.0002**	1.00	Reference	
Health Center	995	298	30.0		0.88	0.68–1.15	0.3581
Home	414	163	39.4		1.31	0.96–1.75	0.0858
Other	205	48	23.4		0.82	0.55–1.22	0.3225
**Age**							
5 years old	980	288	29.4	0.0791	1.00	Reference	
6–7 years old	1039	343	33.0		1.17	0.96–1.42	0.1154
**Mother HAV**							
Positive	585	31.6	0.2331				
Negative	48	27.3					
**Mother age**							
< 29 years old	695	223	32.1	0.6853	1.00	Reference	
30–39 years old	1059	322	30.4		0.82	0.66–1.03	0.0909
≥ 40 years old	265	86	32.5		0.83	0.59–1.16	0.2720
**Mother education**							
No/primary	1462	487	33.3	**0.0040**	1.00	Reference	
Junior High School	435	116	26.7		0.84	0.65–1.09	0.1874
Senior HS/College	122	28	23.0		0.83	0.52–1.31	0.4173
**House roof**							
Tile	501	158	31.5	0.2298	1.00	Reference	
Metal	1271	401	31.6		0.99	0.79–1.25	0.9461
Cement	38	6	15.8		0.40	0.16–1.00	**0.0500**
Other	209	66	31.6		0.93	0.65–1.34	0.7154
**Number of siblings**							
0–2	1049	303	28.9	**0.0170**	1.00	Reference	
≥ 3	970	328	33.8		1.21	0.97–1.50	0.0875
**Father’s Job**							
Fisherman	70	38	54.3	** < 0.0001**	2.45	1.47–4.08	**0.0005**
Other	1949	593	30.4		1.00	Reference	
**Traveling time to health center**							
~ 15 min	977	262	26.8	** < 0.0001**	1.00	Reference	
15–29 min	511	153	29.9		1.17	0.92–1.49	0.2114
30–44 min	353	134	38.0		1.51	1.16–1.98	**0.0023**
45–59 min	150	76	50.7		2.41	1.68–3.47	** < 0.0001**
Never	28	6	21.4		0.51	0.20–1.31	0.1611
N = 2019	*p* < 0.0001						

Bold values are used to highlight the significant results having significant association.

### Discussion

This study represents the first nationwide large-scale sero-epidemiological study on the prevalence of total anti-HAV among 5–7 years old children and their mothers in Cambodia, as of 2017. Detection of total anti-HAV indicates the past or present infection with HAV or vaccination against HAV. Although it cannot clarify them, it shows the previous exposure to HAV naturally or vaccination-mediated and is useful to examine the disease prevalence and its burden.

Considering the period of country's gross development, two cohorts were included in this study; 2021 mothers born before the country's gross development and 2514 children born after the country's gross development. The overall total anti-HAV prevalence among children was 31.5%, and that among mothers was 91.2%. We found a gross difference in total anti-HAV prevalence with a high prevalence profile in mothers and a low profile in children. Considering that mothers had long exposure time to HAV than their children and the impact of characteristics of two different cohorts, we suggested that this difference was due to age effect and cohort effect. However, our study reported the updated estimation on the prevalence of total anti-HAV among children and their mothers as representative of the whole country.

The report in 1993 stated that 27–97% of children and 100% of adults were infected with HAV in Takeo province^9^. Chhour YM et al. reported that 55% of viral hepatitis diagnosed among children admitted to the National Pediatric Hospital as of 1998 were due to HAV^8^. The prevalence of anti-HAV among children of that time reflects the mother cohort of our study when they were in childhood life. By our study, total anti-HAV prevalence among children ranged from 5.6 to 56.9% and that among mothers ranged from 77.6 to 100%. After the country’s improvement in safe water and food supply, hygiene and good sanitation, the prevalence decreased over two decades. However, a reduced anti-HAV prevalence exposes to future epidemics due to the lack of herd immunity among population^13^. This situation is now occurring in the developed countries like Japan. If the hygiene and sanitation are sustainably improved, or the current situation is kept the same in Cambodia, we suggest that the children might not have the same risky environment as their mothers, and the prevalence might be no longer increased when the children become adults. Therefore, it is essential to monitor changes in the prevalence and implement vaccination against hepatitis A if the situation changes significantly.

In our study, the within-country difference in total anti-HAV prevalence was found. Some provinces (Kampong Speu and Kandal) had a high prevalence of total anti-HAV in both mother and children cohort. Meanwhile, some provinces (Koh Kong, Kampot, Takeo, Prey Veng, Svay Rieng, Siem Reap, and Battanbbang) had an intermediate prevalence in the mother cohort but a high prevalence in the children cohort. This finding could suggest that safe food and water supply, hygiene and sanitation were not sufficiently improved in these regions. Moreover, internal migration should be considered, as positive cases may have moved to other areas. Interestingly, all provinces with high prevalence are located either in the seaside or along the Tonle Sap lake, where the majority are fishermen. The fishermen, dealing with the raw fishes everyday, and their lifestyle habitats superimpose the risk of HAV. In addition to the country's gross development, geographical preference with the majority of work, their tradition and lifestyle concomitantly related to the prevalence of HAV infection.

The above finding was supported by the subsequent multivariate analysis. By logistic regression, children living in urban areas and those with cement roof, a proxy indicator of socio-economic status, had the lowest HAV risks. But those whose fathers were fishermen and those living far from the health care center had the highest potential for HAV infection.

Another noteworthy finding of this study was that we used the DBS as the new sampling strategy instead of collecting whole blood samples from all participants. The validity and efficacy of DBS for detection of HBV sero-markers^14^ and also the usefulness of DBS for molecular analysis of HBV^15^ had been reported previously. This study examined the sensitivity and specificity of total anti-HAV detection using DBS-driven samples against gold standard serum samples and set the cutoff value for the DBS-driven samples. The reported sensitivity of 94.8% and the specificity of 99.0% were applicable for the detection of total anti-HAV using DBS-driven samples. This study elucidated the efficacy of DBS as a new sampling tool for large-scale epidemiological studies in resource-limited countries with insufficient infrastructure, human resources, and technology.

There are some limitations in our study. Firstly, we could not identify the acute cases or immune due to vaccination, and HAV vaccination history was also unknown. Moreover, 94.8% sensitivity of the anti-HAV test used in this study may underestimate the true prevalence.

In conclusion, the low prevalence of total anti-HAV among children indicates the country’s improvement of safe water and food supply, hygiene, and sanitation. If the hygiene and sanitation are consistently improved in Cambodia, the prevalence might be no longer increased when the children become adults. Otherwise, there is a potential outbreak in the future due to a lack of herd immunity. The public health sector should consider raising public awareness through hygiene promotion and preventive measures.

## Data Availability

All the data included in this study were fully described in the manuscript.
